# COX7AR is a Stress-inducible Mitochondrial COX Subunit that Promotes Breast Cancer Malignancy

**DOI:** 10.1038/srep31742

**Published:** 2016-08-23

**Authors:** Kezhong Zhang, Guohui Wang, Xuebao Zhang, Philipp P. Hüttemann, Yining Qiu, Jenney Liu, Allison Mitchell, Icksoo Lee, Chao Zhang, Jin-sook Lee, Petr Pecina, Guojun Wu, Zeng-quan Yang, Maik Hüttemann, Lawrence I. Grossman

**Affiliations:** 1 Center for Molecular Medicine and Genetics, Wayne State University, Detroit, MI 48201, USA; 2 Department of Immunology and Microbiology, Wayne State University School of Medicine, Detroit, MI 48201, USA; 3 Karmanos Cancer Institute, Wayne State University School of Medicine, Detroit, MI 48201, USA; 4 Department of Internal Medicine, The Affiliated Tumor Hospital of Zhengzhou University, 127 Dongming Road, Jinshui, Zhengzhou, Henan, 450008, China; 5 College of Medicine, Dankook University, Cheonan-si, Chungcheongnam-do 330-714, Republic of Korea; 6 Department of Biochemistry and Molecular Biology, Wayne State University School of Medicine, Detroit, MI 48201, USA.

## Abstract

Cytochrome *c* oxidase (COX), the terminal enzyme of the mitochondrial respiratory chain, plays a key role in regulating mitochondrial energy production and cell survival. COX subunit VIIa polypeptide 2-like protein (COX7AR) is a novel COX subunit that was recently found to be involved in mitochondrial supercomplex assembly and mitochondrial respiration activity. Here, we report that COX7AR is expressed in high energy-demanding tissues, such as brain, heart, liver, and aggressive forms of human breast cancer cells. Under cellular stress that stimulates energy metabolism, COX7AR is induced and incorporated into the mitochondrial COX complex. Functionally, COX7AR promotes cellular energy production in human mammary epithelial cells. Gain- and loss-of-function analysis demonstrates that COX7AR is required for human breast cancer cells to maintain higher rates of proliferation, clone formation, and invasion. In summary, our study revealed that COX7AR is a stress-inducible mitochondrial COX subunit that facilitates human breast cancer malignancy. These findings have important implications in the understanding and treatment of human breast cancer and the diseases associated with mitochondrial energy metabolism.

Biogenesis of eukaryotic COX involves the coordinated action of the mitochondrial DNA-encoded subunits, which form the catalytic core of the enzyme, and the nuclear DNA-encoded subunits, which play a largely unknown but presumed regulatory and possibly protective role^1^,^2^. Regulation of COX biogenesis and activity in response to changing environmental or physiological conditions is critical for stress-adapting cells to make survival or death decisions. To adjust the production of energy to the variable energetic requirements of the cell, mitochondrial respiration is tightly regulated through modulating expression, import, and assembly of the COX subunits^1^,^3^. Such regulation ensures the building of a highly efficient molecular machine, able to catalyze the transfer of electrons from cytochrome *c* to molecular oxygen and ultimately to facilitate aerobic production of ATP. However, the mechanisms by which COX subunits modulate COX activity and ATP production under stress conditions remain to be elucidated.

COX7A is one of 10 nuclear-encoded subunits of the COX holoenzyme, and one of six that have isoforms with tissue-specific differences in expression. A new member of the COX7A gene family, *COX7AR* (also called * COX7A2L, SIG81*, and *COX7RP*), was previously identified from both a mouse silica-induced gene library and human expressed sequence tags^4^. This gene was found to respond to estrogen^5^ and was shown to resemble both standard forms, *COX7AL (COX7A2)* and *COX7AH (COX7A1)*, especially with respect to a 13-residue “functional core” of the mammalian protein previously identified by comparisons between *COX7AL* and *COX7AH*^2^,^6^,^7^. Amino acids of COX7AR are evolutionarily conserved across mammalian species^6^, suggesting that *COX7AR* is of functional importance. Recent research suggested that COX7AR may function as a stabilizing factor that promotes mitochondrial supercomplex assembly and is required for full activity of mitochondrial respiration^8^,^9^.

In this study, we investigated the regulatory mechanism of COX7AR and its potential role in cancer cell malignancy. Our work revealed that COX7AR acts as a stress-inducible COX subunit that plays a role in facilitating human breast cancer growth and expansion. The findings from our study contribute to the understanding of cancer oncogenesis and possibly other human diseases associated with mitochondrial metabolism under stress conditions.

## Results

### COX7AR is highly expressed in human breast cancer cells and inducible by cellular stress

**Figure 1:**
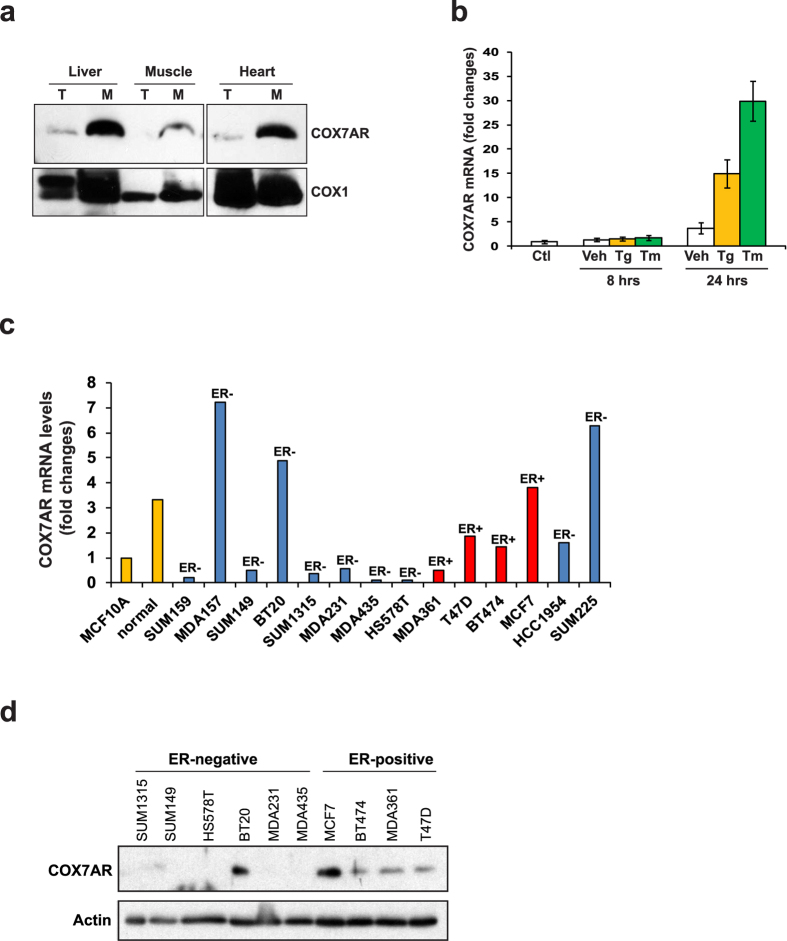
(**a**) Western blot analysis of COX7AR in mouse liver, muscle and heart tissues. T, total cell lysates; M, mitochondrial protein fraction. (**b**) Expression of *Cox7ar* mRNA in mouse embryonic fibroblasts without (Ctl) or with the treatment of vehicle (Veh), thapsigargin (Tg, 0.2 μM), or tunicamycin (Tm, 5 μg/ml) for 8 and 24 h. Fold-change of mRNA was determined by quantitative real-time PCR. (**c**) Quantitative real-time PCR analysis of *COX7AR* mRNA levels in estrogen receptor (ER)-positive or -negative human breast cancer cell lines. Expression values were normalized to β-actin. Fold changes of mRNA levels are shown by comparison to that of MCF10A cells. (**d**) Western blotting analysis of COX7AR protein expression in some representative breast cancer cell lines. β-actin was included as a protein loading control.

COX7AR was originally identified from both a mouse silica-induced gene library and human expressed sequence tags^4^. However, expression of COX7AR was barely detectable in many transformed cell lines or tissues (data not shown). Genome-wide transcription profile analysis suggested that expression levels of human *COX7AR* mRNA are higher in secretory, high-energy-demanding cells, compared to other cell types^10^,^11^. To evaluate tissue-specific expression of the COX7AR protein, we analyzed levels of COX7AR in total cellular protein lysates and in mitochondria-enriched protein fractions isolated from various mouse tissues. Through Western blot analysis, we detected relatively high levels of COX7AR protein in the mitochondria-enriched protein fractions isolated from mouse liver, muscle, and heart tissues (Fig. 1a). To test whether expression of COX7AR is cell stress-inducible, we treated mouse embryonic fibroblasts (MEFs), which express very low levels of endogenous COX7AR, with the stress-inducing reagents thapsigargin (Tg) or tunicamycin (TM) (Fig. 1b). Tg can trigger calcium release from the endoplasmic reticulum (ER), raising the cytosolic calcium concentration and stimulating mitochondrial metabolism^12^. TM can disrupt protein N-linked glycosylation, causing the accumulation of unfolded proteins in the ER and subsequent activation of ER stress response. We found that both Tg and TM treatment significantly up-regulates expression of *COX7AR* mRNA in MEFs (Fig. 1b), suggesting that expression of the *COX7AR* gene is stress-inducible. Further, we examined expression of COX7AR in representative human breast cancer cell lines that are commonly used as tumor models. These cell lines include several breast cancer subtypes characterized by the tumor grades and the presence of estrogen receptor (ER), progesterone receptor (PR) and Her-2 (ERBB2) as classifiers^13^,^14^,^15^: (i) the immortalized but nontransformed human mammary epithelial cell line MCF10A; (ii) the triple-negative (ER-, PR- and Her2-negative) breast cancer cell lines SUM159, SUM149, MDA-157 and BT20; (iii) the Her2-positive breast cancer cell lines SUM225 (ER−) and HCC1954 (ER−); (iv) the ER-negative breast cancer cell lines with intermediate response to chemotherapy, including MDA-231, Hs578T, SUM1315, and MDA435; and (v) the Luminal (ER+) breast cancer cell lines MCF-7, T47D, BT474, and MDA361. Quantitative real-time PCR (qRT-PCR) analysis indicated that expression of endogenous *COX7AR* mRNA was significantly induced in both ER-positive and ER-negative human breast cancer cell lines compared to those in normal human mammary gland epithelial cells or non-aggressive breast cancer cell lines (Fig. 1c). Although a previous report suggested that expression of COX7AR was inducible by estrogen signals^5^, the qRT-PCR result indicated that COX7AR is also highly induced in some ER-negative, highly-malignant breast cancer cell lines, such as the triple-negative breast cancer cell lines BT20 and MDA-157 (Fig. 1c). Additionally, Western blot analysis confirmed expression of COX7AR protein in some representative ER-positive and ER-negative breast cancer cell lines (Fig. 1d).

### COX7AR is present in the mitochondrial COX complex during cellular stress

**Figure 2:**
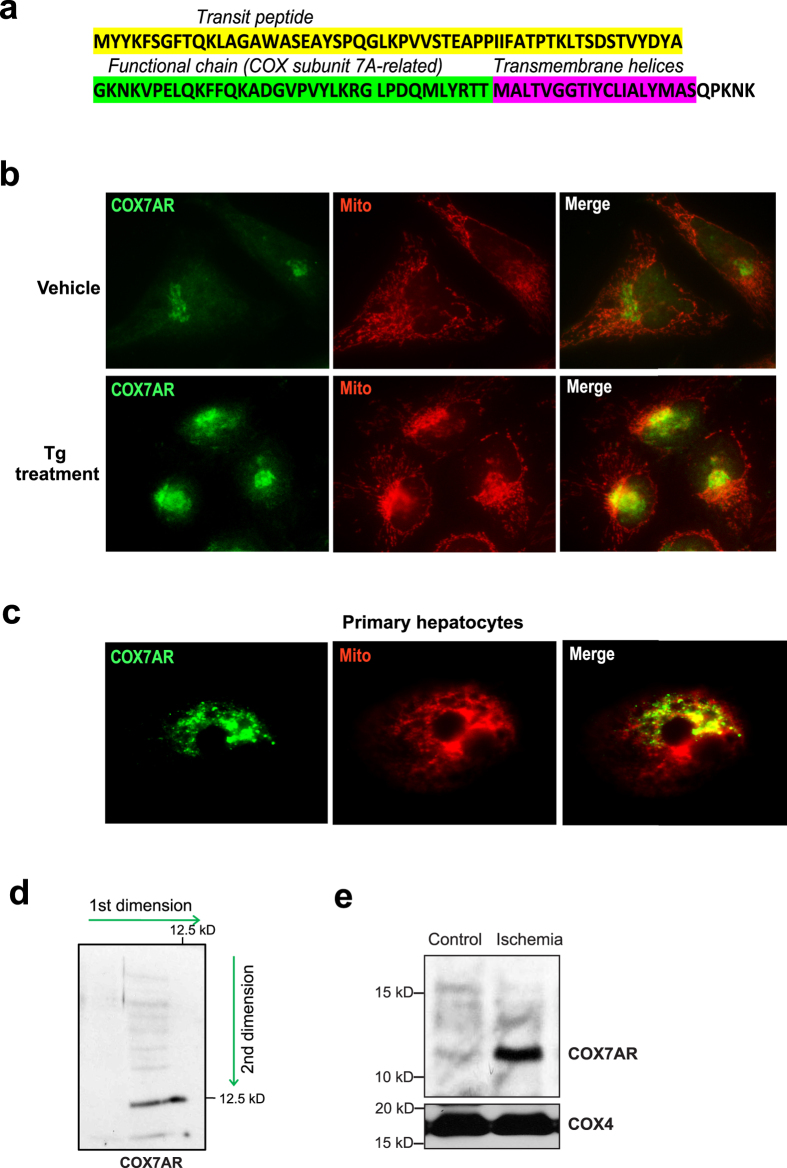
(**a**) Protein sequence analysis for human COX7AR protein. (**b**) Immunofluorescent analysis for COX7AR localization before and after Tg (0.2 μM) treatment for 8 h. CHO cells were transfected with plasmids expressing flag-tagged human COX7AR. Cells were stained with MitoTracker Red for mitochondria. COX7AR protein was detected via green fluorescence (Magnification ×600). (**c**) Immunofluorescent analysis for COX7AR localization in mouse primary hepatocytes. Cells were stained with MitoTracker Red for mitochondria. The endogenous COX7AR protein was stained with the anti-COX7AR antibody for green fluorescence (Magnification ×600). (**d**) 2-D Western blot analysis of COX7AR in mitochondria-enriched mouse liver protein fractions. (**e**) Presence of COX7AR in the COX enzyme purified from bovine heart without (control) or with 1 h of ischemic stress (ischemia) was detected by Western blot with an anti-COX7AR antibody. COX subunit 4 was used as loading control.

As shown by protein sequence analysis, the human COX7AR protein contains a transit peptide, a functional chain, and transmembrane helix (Fig. 2a). Interestingly, the N-terminal transit peptide is inconsistent with traditional mitochondrial targeting sequences^7^. It is possible that the transit peptide can be further processed into a mature form under stress conditions, which may then lead to translocation of COX7AR into the mitochondria. To determine the subcellular localization of COX7AR under cellular stress, CHO cells expressing flag-tagged human COX7AR were treated with Tg (0.2 μM) or vehicle for 8 h before immunofluorescent staining of COX7AR protein (green fluorescence) and mitochondria (red fluorescence). After Tg treatment, a portion of COX7AR translocated to the mitochondria, as revealed by the merged fluorescence signals (Fig. 2b). Similar results were found when cells were treated with TM (data not shown). Further, we examined the subcellular localization of COX7AR in primary mouse liver hepatocytes, a mitochondria-enriched, metabolic cell type. A significant amount of COX7AR proteins were expressed in primary hepatocytes, and a majority of these COX7AR proteins were localized in the mitochondria (Fig. 2c). The presence of COX7AR protein in the liver mitochondria compartment was confirmed by 2-D Western blot analysis with the mitochondria-enriched mouse liver protein fractions (Fig. 2d). Another type of severe stress that affects mitochondrial function is ischemia as seen in myocardial infarction. To test the effect of ischemia, we examined the presence of COX7AR in the COX enzyme complex purified from cow heart tissues under the ischemic condition^16^. COX7AR was barely detectable in control COX purified from the heart tissue that was immediately frozen on dry-ice after the animals were sacrificed (Fig. 2e). However, when the heart was kept at 37 °C for 1 h in the absence of oxygen to simulate ischemia during myocardial infarction, levels of COX7AR present in the purified COX complex were significantly increased (Fig. 2e), suggesting that increased incorporation of COX7AR in the COX enzyme complex is driven by ischemic stress. Together, these results suggest that a portion of COX7AR is localized within mitochondria in mitochondria-enriched metabolic cell types and that cellular stress can induce COX7AR translocation to the mitochondria and incorporation into the COX holoenzyme.

### COX7AR promotes cellular energy production upon cellular stress

**Figure 3:**
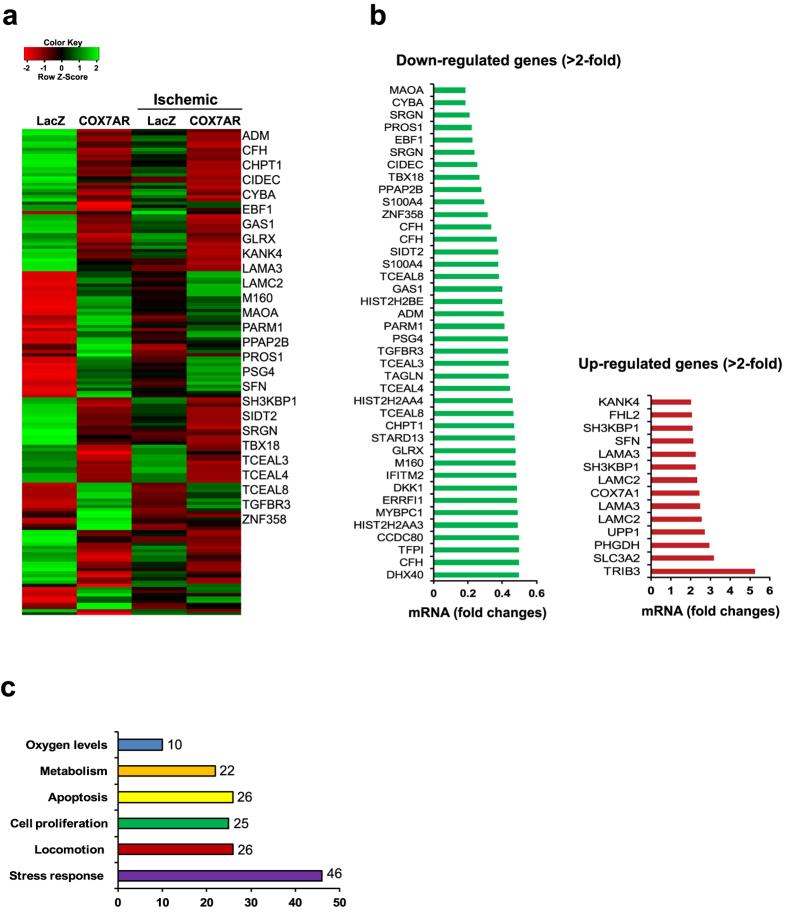
Microarray analysis with COX7AR- or LacZ-expressing MCF10A stable cell lines under ischemic/hypoxic stress. COX7AR- or LacZ-expressing MCF10A stable cell lines were cultured under the non-stressed or ischemic/hypoxic stress for 90 min before isolation of total RNA followed by Illumina microarray analysis. (**a**) The microarray heatmap and top 27 functionally characterized genes whose expression were significantly altered in the COX7AR-expressing cells, compared to those in the LacZ-expressing cells under control or ischemic/hypoxic stress condition. (**b**) Fold changes of the transcripts encoded by the genes that were up- or down-regulated in the COX7AR-expressing MCF10A cells under the non-stressed or ischemic/hypoxic stress conditions. As described in the method session, False Discovery Rate (FDR) test was used for multiple corrections (cut-off p-value ≤ 0.05) and a fold change criterion (cut-off value >2) to determine differential gene expression. The genes whose expression was significantly altered between COX7AR- and LacZ-expressing MCF10A cells were included. (**c**) Functional clusters of genes whose expression were significantly altered in the MCF10A cells upon expression of exogenous COX7AR.

To determine the functional involvement of COX7AR, we performed gene expression microarray analyses with the COX7AR-expressing human mammary gland epithelial cell line MCF10A in the presence or absence of ischemic stress. We generated a MCF10A cell line that stably expresses COX7AR or LacZ as a control. The MCF10A cell lines were challenged with ischemic/hypoxic stress by incubating the cells for 90 min in a 0.2% oxygen chamber using an ischemia mimetic solution that lacks glucose as previously described^17^. Microarray analysis indicated that gene expression profiles in MCF10A cells and expression of *COX7AR* were altered in the presence of ischemic stress (Fig. 3a). Bioinformatics analysis revealed 39 genes whose expression levels were significantly reduced and 14 genes whose expression levels were significantly increased in the COX7AR-expressing MCF10A cells (Fig. 3b). The majority of the genes whose expression levels were significantly altered are involved in stress response, cell proliferation, and stress-induced apoptosis (Fig. 3c). Among these genes, 46 genes are involved in stress response, 25 genes are involved in cell proliferation, 26 genes are involved in locomotion, 26 genes are involved in apoptosis, 22 genes are involved in metabolism, and 10 genes are involved in cellular oxygen levels. Interestingly, the most upregulated gene, *TRIB3*, is a disease-related gene that has been called a “stress adjusting switch”^18^.

**Figure 4:**
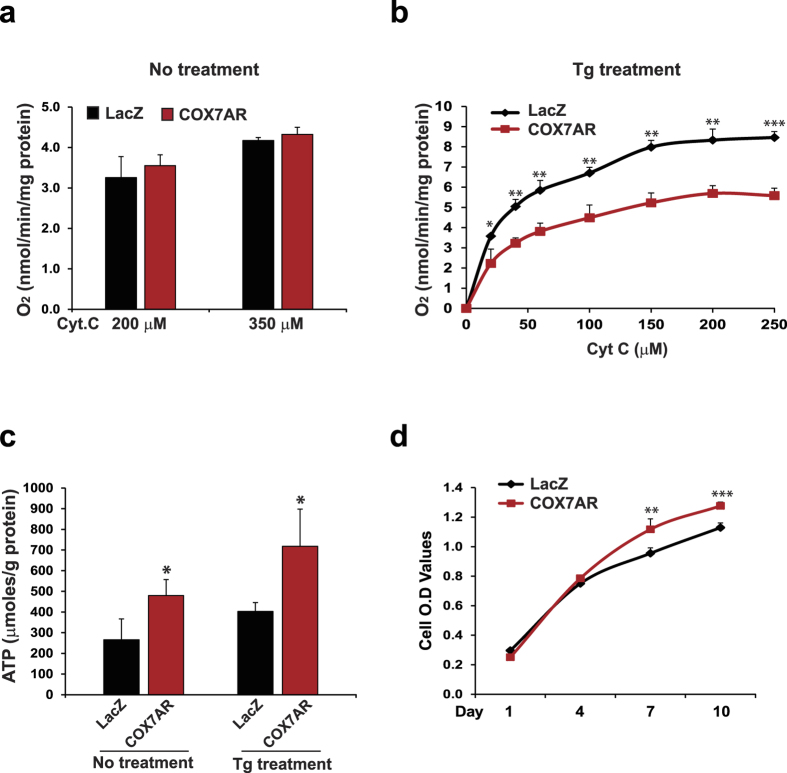
COX activities and ATP levels in the COX7AR- or LacZ-expressing MCF10A stable cell lines under non-stressed conditions or after Tg (0.5 μM) treatment for 6 h. (**a,b**) COX specific activities, shown as O_2_ consumption rate after titrating with increasing amounts of substrate cytochrome *c* (Cyt C) in solubilized COX7AR- or LacZ-expressing MCF10A cells under non-stressed conditions (**a**) or after 6-h Tg treatment (**b**). Data are shown as mean ± SEM (n = 3 biological repeats). *P ≤ 0.05; **P ≤ 0.01; ***P < 0.001. (**c**) Amounts of ATP produced by the COX7AR- or LacZ-expressing MCF10A cells under the non-stressed condition or after 6-h Tg treatment. (**d**) Proliferation rates of COX7AR- or LacZ-expressing MCF10A stable cell lines determined by Cell Titer Aqueous Cell Proliferation Assay kit (Promega). Data are shown as mean ± SEM (n = 4 biological repeats). **P ≤ 0.01; ***P < 0.001.

The microarray analysis result suggested that COX7AR, as a stress-induced mitochondrial component, might be functionally involved in energy metabolism. To test this possibility, we examined COX activity and ATP production in COX7AR- or LacZ-expressing MCF10A cells under the non-stressed condition or upon Tg treatment that stimulates mitochondrial metabolism. The COX7AR- or LacZ-expressing stable MCF10A cell lines were cultured in the presence or absence of Tg for 6 h following determination of COX-specific activities or ATP levels. Although there was no significant difference in COX activity, reflected by the consumption of oxygen (O_2_) in the presence of cytochrome *c*, between the COX7AR-expressing and the control MCF10A cells under the non-stressed condition (Fig. 4a), COX activity of the COX7AR-expressing MCF10A cells was significantly lower than that of the control cells upon the Tg treatment (Fig. 4b). Conversely, the COX7AR-expressing cells produced significantly higher levels of ATP than the control cells under either the non-stressed condition or in response to Tg treatment (Fig. 4c). It is possible that the MCF10A cell line was transformed by stable expression of exogenous COX7AR, and therefore, becomes more cancerous and exhibits the Warburg effect^19^, as reflected by lower COX activity upon cellular stress (Fig. 4b). Since glycolysis operates faster than oxidative phosphorylation, high ATP levels (Fig. 4c) can be maintained as long as glucose does not become limiting. Nevertheless, these results confirmed that COX7AR promotes cellular energy production, especially under the stress condition that stimulates mitochondrial metabolism. Furthermore, supporting the role of COX7AR in promoting cellular energy production, the proliferation rate of COX7AR-expressing MCF10A cells was significantly higher than that of the control MCF10A cells expressing LacZ (Fig. 4d).

### COX7AR facilitates breast cancer cell growth, clone formation, and invasion

**Figure 5:**
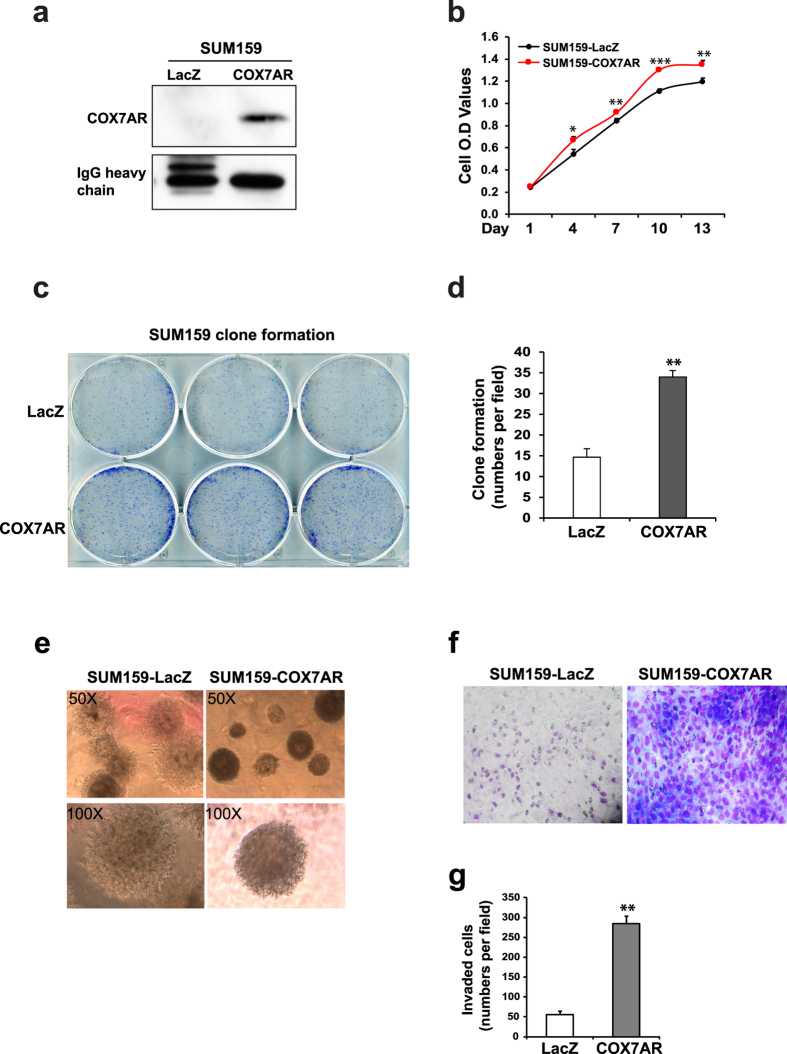
(**a**) IP-Western blot analysis of the COX7AR levels in the stable human breast cancer cell line SUM159 that was transduced by lentivirus expressing COX7AR or LacZ. Total cellular lysates were subjected to pull-down using the anti-COX7AR antibody to enrich for COX7AR protein, followed by Western blot analysis using the same antibody. The levels of IgG heavy chain were included as loading controls. (**b**) Proliferation rates of COX7AR- or LacZ-expressing SUM129 stable cell lines determined by Cell Titer Aqueous Cell Proliferation Assay kit (Promega). Data are shown as mean ± SEM (n = 4 biological repeats). *P ≤ 0.05; **P ≤ 0.01; ***P < 0.001. (**c**) Clone formation of the COX7AR- or LacZ-expressing SUM129 cells in Matrigel. (**d**) Quantification of clone formation by SUM159-LacZ and SUM159-COX7AR cells in Matrigel. The clone numbers in a random field of the same size per sample were calculated. Data are shown as mean ± SEM (n = 3 biological repeats). **p ≤ 0.01. (**e**) Morphology of SUM159-LacZ and SUM159-COX7AR cell clones in Matrigel (Magnification is ×400). (**f**) Invasion assay of the COX7AR- or LacZ-expressing SUM129 cells in Matrigel. Experiments were repeated three times, and representative images are shown. (**g**) Quantitative analysis of SUM159-LacZ and SUM159-COX7AR cell invasion in Matrigel. The invaded cell numbers in a random field of the same size per sample were calculated. Data are presented as means ± SEM (n = 5 biological repeats). **P < 0.01.

**Figure 6:**
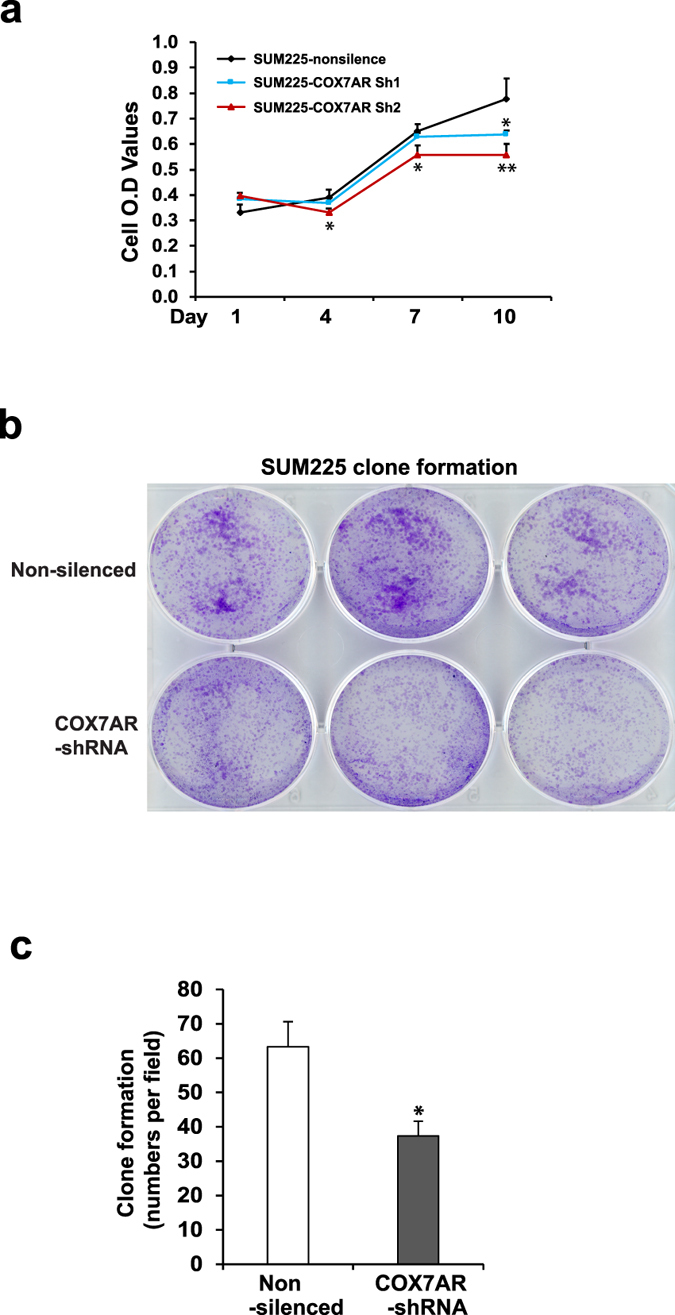
(**a**) Proliferation rates of COX7AR-knockdown and control SUM225 stable cell lines were determined by Cell Titer Aqueous Cell Proliferation Assay kit (Promega). Two COX7AR-knockdown SUM225 stable cell lines were generated by transducing lentivirus expressing COX7AR shRNA. SUM225 cells were transduced with lentivirus expressing non-silencing shRNA as the control. Data are shown as mean ± SEM (n = 3 biological repeats). *p ≤ 0.05; **p ≤ 0.01. (**b**) Clone formation of the COX7AR-knockdown or non-silencing shRNA control SUM225 cells in Matrigel. (**c**) Quantification of clone formation by nonsilencing SUM225 control and COX7AR-knockdown SUM225 cells in Matrigel. The clone numbers in a random field of the same size per sample were calculated. Data are shown as mean ± SEM (n = 3 biological repeats). *p ≤ 0.05.

Prompted by the Warburg-like phenotype, we performed gain- and loss-of-function studies to elucidate the role of COX7AR in cancer cell proliferation and malignancy. First, we introduced exogenously expressed COX7AR in the human breast cancer cell line SUM159, in which endogenous COX7AR was barely detectable, and established a stable COX7AR-expressing breast cancer cell line, SUM159-COX7AR (Fig. 5a). Cell proliferation analysis showed that SUM159-COX7AR cells displayed increased proliferation rates compared to SUM159-LacZ control cells (Fig. 5b). Further, we evaluated the effects of COX7AR expression on breast cancer cell clone formation and invasion. Anchorage-independent growth assays indicated that the COX7AR-expressing SUM159 cell line formed more clones than the SUM159-LacZ cell line after 21 days of culture (Fig. 5c,d). The SUM159-COX7AR cells displayed the characteristics of dense, round, and smaller sizes, compared to the SUM159-LacZ cells (Fig. 5e). Next, we performed cell invasion assays using a Matrigel invasion chamber. At 48 h after cell seeding, the number of invading SUM159-COX7AR cells was significantly higher than that of SUM159-LacZ invading cells (Fig. 5f,g). We knocked down COX7AR in a human breast cancer cell line, SUM225, which expresses relatively high levels of endogenous COX7AR, by using lentiviral-based COX7AR shRNAs. We established two stable COX7AR-knockdown cell lines, SUM225-COX7AR-shRNA1 and SUM225-COX7AR-shRNA2. Cell proliferation analysis indicated that both knockdown cancer cell lines showed reduced proliferation rates compared to the control cancer cell line expressing the non-silencing vector (Fig. 6a). Clonogenic assays showed that knockdown of COX7AR decreased the numbers of SUM225 clones formed after 21 days of culture (Fig. 6b,c). Taken together, the gain- and loss-of-function analyses suggested that COX7AR promotes human breast cancer cell proliferation, clone formation, and invasiveness.

## Discussion

In this study, we demonstrated that COX7AR is a stress-inducible, mitochondria-associated protein that is involved in human breast cancer cell proliferation and malignancy. COX subunit 7A is one of several nuclear encoded subunits with tissue-specific isoforms. COX7AR resembles both COX7AL and COX7AH with respect to a 13-residue “functional core” of the mammalian mitochondrial protein previously identified by comparisons between *COX7AL* and *COX7AH*^20^. However, the function and subcellular localization of COX7AR were subjects of ambiguity until recent studies suggested that COX7AR may function as a stabilizing factor that promotes mitochondrial supercomplex assembly and is required for full activity of mitochondrial respiration^8^,^9^. Our data, based on cultured cells, suggest that respiration is inversely correlated with *COX7AR* expression (Fig. 4b), implying that additional mechanisms act on COX^21^. Our study suggests that COX7AR is a stress-inducible COX subunit that is required for metabolic regulation and maintenance of the Warburg effect associated with cancer malignancy. *In vivo*, we found that COX7AR is expressed in high energy-demanding cells and tissues, such as liver, heart, and human breast cancer cells. In culture, expression of COX7AR is inducible by reagents that increase calcium signals and/or cause intracellular stress. In addition, under pathophysiological stress, such as hypoxia, ischemia, and calcium depletion, COX7AR protein is enriched in the mitochondria of the stressed cells. Although COX7AR is hardly detectable in the COX enzyme complex purified from bovine heart tissue under normal conditions, it is present in the purified COX complex from the heart after ischemia. The gain- and loss-of-function analyses demonstrated that COX7AR plays a critical role in maintaining higher proliferation rates, clone formation, and invasion of human breast cancer cells under oncogenesis-associated stress. All these observations support the notion that COX7AR is a stress-inducible COX subunit that adapts metabolism to conditions that interfere with cellular energy metabolism. The COX7AR-mediated stress response may provide an important survival mechanism for cancer cells under oncogenic stress conditions.

Regulation of cellular energy production through the mitochondrial electron transport chain is closely relevant to the stress conditions associated with mitochondrial diseases as well as a number of other energy-related diseases, such as cardiovascular disease, neurodegenerative disease, metabolic syndrome, and cancer. COX is a central player in regulating aerobic energy production. The increased ATP production (Fig. 4c) occurring in the face of decreased COX activity (Fig. 4b) was seen previously in a COX-caused cardiomyopathy^22^ and may represent a short-term adaptive response in stress-induced mitochondrial hyperfusion^23^,^24^.

Oncogenic stress, which is partially caused by hypoxic conditions and increased growth signaling, results in a shift from energy production primarily relying on oxidative phosphorylation to primarily relying on glycolysis^19^. Therefore, high induction of COX7AR in human breast cancer cells, especially aggressive ones (Fig. 1c), is consistent with the role of COX7AR as a stress-inducible COX subunit that facilitates cancer cell proliferation and clone formation. Delineation of the regulatory network associated with COX7AR may increase our understanding of the fundamental process and physiological significance of signal transduction in cellular energy metabolism.

## Materials and Methods

### Origins of biochemical materials, animal tissues and human breast cancer cells

Chemicals were purchased from Sigma unless indicated otherwise. Synthetic oligonucleotides were purchased from Integrated DNA Technologies, Inc. (Coralville, IA). The rabbit anti-COX7AR polyclonal antibody was purchased from Protein Tech, Inc. (Chicago, IL). The experiments with animal tissue samples were approved by the Wayne State University Institutional Animal Care and Use Committee (IACUC) and carried out under the institutional guidelines for ethical animal use. The previously-established human breast cancer cell lines SUM159, SUM149 and SUM225 as well as MCF10A^25^ were obtained from Dr. Stephen P. Ethier at the Medical University of South Carolina, Charleston, SC, USA. All the other breast cancer cell lines were purchased from ATCC and were authenticated in the Biobanking and Correlative Sciences Core at Karmanos Cancer Institute.

### Culture of the cell lines used in this study

The SUM225 cell line was established from a chest wall recurrence of ductal carcinoma *in situ* of the breast, and the SUM159 cell line was established from an invasive ductal breast carcinoma^25^. SUM225, SUM149, and SUM159 cells were cultured with 5% fetal bovine serum, fungizone (0.5 μg/mL), gentamicin (5 μg/mL), hydrocortisone (1 μg/mL), and insulin (5 μg/mL). MCF10A, a spontaneously immortalized but nontransformed human mammary epithelial cell line, and the other human breast cancer cell lines utilized in this study, including SUM1315, MDA435, MDA231, HS578T, MDA361, T47D, BT474, MCF7, MDA157, HCC1954 and BT20, were cultured as previously described^26^,^15^. Primary hepatocytes from wild-type C57BL/6J mouse livers were isolated and cultured as previously described^27^.

### Construction of plenti-COX7AR lentivirus

The human *COX7AR* cDNA was amplified from HEK293 cells and verified by DNA sequencing. The primers used for COX7AR gene cloning were forward primer 5′-CACCATGTACTACAAGTTTAGTGGC-3′, and reverse primer 5′-TTTGTTTTTGGGCTGCGAAG-3′. Full-length human COX7AR cDNA was sub-cloned into pENTR/directional vector. The *COX7AR* cDNA was then recombined into the pLenti6/V5-DEST vector with LR Clonase II enzyme. 293FT producer cells were co-transfected with 3 μg pLenti expression plasmid DNA and 9 μg of ViraPower packaging mix using the Lipofectamine-2000 reagent (Invitrogen, Carlsbad, CA, USA). Lentivirus containing supernatants were collected after 48 h, filtered through 0.45 μm PVDF filters (Millipore), and then used to infect MCF10A, SUM159, or CHO-K1 cells. Selection of stably transduced cell lines was started at 48 h after infection with 10 μg/ml Blasticidin (Invivogen, San Diego, CA).

### Construction of *COX7AR* microRNA-adapted shRNA (shRNAmir) knockdown lentivirus

pGIPZ-based short hairpin RNA (shRNA) lentiviral vectors were purchased from the Open Biosystems (clone VGH5518-98713873 and VGH5518-99293250). The vectors were packaged by cotransfection of 293T cells with five packaging plasmids (pTLA1-Pak, pTLA1-Enz, pTLA1-Env, pTLA1-Rev, and pTLA1-TOFF) by using the Trans-Lentiviral™ Packaging System (Open Biosystems). Lentivirus containing supernatants were collected 48 h after transfection and filtered with 0.45 μm PVDF filters (Millipore) before they were used to infect human breast cancer cells. Stable populations of lentivector-transduced cells were selected by culture in puromycin (10 μg/ml). Knock-down efficiency was confirmed by examining expression of *COX7AR* transcripts in the SUM225 cells after a 2-week selection.

### Immunofluorescence assay

Indirect immunofluorescence was performed as previously described^28^. In brief, cells were grown on glass coverslips, fixed with 4% paraformaldehyde in PBS followed by blocking with 5% normal goat serum in 0.1 Triton X-100/PBS before incubation with primary antibodies. Secondary antibodies used were Alexa Fluor 594- or Fluor 488-conjugated anti-rabbit IgG (Invitrogen). Slides were mounted with Gold anti-fade reagent. Fluorescent signals were observed and photographed using a fluorescence microscope.

### Isolation of cytochrome *c* oxidase (COX) enzyme

Mitochondrial COX enzyme was isolated from cow heart tissue as previously described^29^. Tissue (250 g) was homogenized for isolating COX-containing fractions. Fractionation of mitochondrial COX proteins was performed with 28% ammonium sulfate for at least 6 h. Proteins were collected by centrifugation for 15 min at 27,000 × *g*. Precipitated proteins were dissolved in 50 mL KH_2_PO_4_ (pH 7.4) and stored at −80 °C after determination of COX concentration and purity by spectrophotometer.

### Cell proliferation assay

For cell proliferation assay, stably transduced or control SUM159, SUM225, or MCF10A cell lines were seeded in triplicate at a density of 2.5 × 10^3^ per well in 96-well plates on day 0. Cell proliferation rates were determined using the CellTiter 96 non-radioactive cell proliferation *assay* (*MTT*) kit from Promega (Madison, WI). After cell culture for 1, 4, 7, 10, or 13 days, 20 μl of MTT (5 mg/ml) solution were added to 200 μl medium in each well. Cells were cultured for an additional 3 h before measuring O.D. values using a plate reader.

### Western blot and IP-Western blot analyses

To determine expression protein levels of COX7AR, CHOP, XBP1, α-tubulin, or GAPDH, total cell lysates were prepared from cultured cells or liver tissue using NP-40 lysis as previously described^30^. Denatured proteins were separated by SDS-PAGE on 10% Tris-glycine polyacrylamide gels and transferred to a 0.45 μm PVDF membrane, followed by probing with anti-COX7AR (Protein Tech, Chicago), anti-CHOP (Santa Cruz Biotech), anti-XBP1 (Santa Cruz Biotech), anti-α-tubulin (Sigma), or anti-GAPDH (Sigma) antibodies. Detection was performed using enhanced chemiluminescence detection reagent. For IP-Western blot analyses, total protein lysates from cultured cells were immunoprecipitated with an anti-V5 antibody (Invitrogen), followed by Western blot analysis with the anti-COX7AR antibody.

### Quantitative real-time PCR analysis

For quantitative real-time PCR analysis, total cellular RNA was prepared using TRIzol reagent (Invitrogen), and reverse-transcribed to cDNA using random primers. The real-time PCR reaction mixture containing cDNA template, primers, and SYBR Green PCR Master Mix (Invitrogen) was run in a 7500 Fast Real-time PCR System (Applied Biosystems, Carlsbad, CA). The sequences of real-time PCR primers used to detect human COX7AR mRNA were forward primer 5′-AAACTGACCTCCGATTCCAC-3′ and reverse primer 5′-AGTAGATGGTCCCTCCCACA-3′. Fold changes of mRNA levels were determined after normalization to β-actin RNA levels as internal control.

### Microarray analysis

COX7AR- or LacZ-expressing MCF10A cells were challenged with ischemic/hypoxic stress by incubating the cells in ischemia mimetic solution lacking glucose for 90 min in a 0.2% oxygen chamber for 90 min as previously described^17^. Total RNAs were isolated from the cells after the treatment and subjected to Illumina Microarray analysis. The “Lumi” package of R software was utilized for data preprocessing, including background correction, variance stabilization transform, quantile normalization correction, and quality control evaluation for raw data^31^. An empirical Bayes method was utilized to detect significant genes identified by using the “limma” package of R^32^. A False Discovery Rate (FDR) test was used for multiple corrections (cut-off p-value = 0.05) and a fold change criterion (cut-off value = 2) to determine differential gene expression. The data were imported using the “gplot” package of R to create heat maps^33^. We next compared the genes whose expression was significantly altered between COX7AR- and LacZ-expressing MCF10A cells in both stressed and non-stressed groups.

### Measurement of COX specific activity and cellular ATP levels

MCF10A cells stably expressing COX7AR or LacZ were cultured in Ham’s F12 medium supplemented with 0.1% BSA, insulin and epidermal growth factor as previously described^34^. When the cells reached 90% confluency, they were treated with PBS or 0.5 μM thapsigargin (Tg) for 6 h. COX specific activity of cell lysates was determined as described^29^ after sonication in solubilization buffer (10 mM Hepes, 40 mM KCL, 1% Tween-20, 2 mM EGTA, 1 mM Na-vanadate, 2 μM Oligomycin, 1 mM PMSF) in a closed Clark-type oxygen electrode chamber (Hansatech Instruments, Ltd., Norfolk, UK) at 25 °C by increasing the amount of bovine heart cytochrome *c* up to 250 μM. ATP levels were determined via the bioluminescent method (HS II kit, Roche Applied Science) in conjunction with the boiling method as described^35^.

### Clone formation assay

SUM225 and SUM159 cell lines and their derived cell lines were seeded in 6-well plates with a density of 5,000 cells/well in the presence of Blasticidin (10 μg/ml) (Invivogen, San Diego, CA) for 2–3 weeks. The same experimental setting was applied to both SFIHE and SFIH culture media. For analysis, cells were stained with crystal violet and the number of transformed foci was determined. To assess anchorage-independent growth, triplicate samples of 5 × 10^4 ^cells from each cell line were mixed 4:1 (v/v) with 2.0% agarose in growth medium to a final concentration of 0.3% agarose. The cell mixture was plated on top of a solidified layer of 0.5% agarose in growth medium (SFIHE). Cells were fed every 3 days with growth medium (SFIHE). Cells were stained with 0.02% iodonitrotetrazolium chloride (Sigma-Aldrich, St. Louis, MO) and photographed after 21 days. Colonies in the entire well were counted using a dissecting microscope and colonies larger than 50 μm were included. Crystal violet staining was used to count the colonies after the 21-day period. Experiments were performed in duplicate and repeated three times.

### Morphogenesis assay

The three-dimensional culture of SUM159 and SUM225 cell lines as well as their derived cell lines on basement membrane was carried out as described previously with minor modifications^34^. Briefly, 5 × 10^3 ^cells were resuspended in modified growth medium (5% IH medium) containing 2% Matrigel (BD Biosciences, San Jose, CA), and seeded on top of a layer of Matrigel. Medium containing 2% Matrigel was added every 3 days. Photographs of representative fields were taken on day 10 or 13.

### Invasion assay

Cell invasion assay was performed using the 24-well Matrigel invasion chamber according to the manufacturer’s instructions (BD Biosciences, San Jose, CA). SUM159 human breast cancer cells were seeded at a density of 2.5 × 10^4^/chamber in SFIH medium. SFIHE medium with 10% FBS was used as a chemoattractant. Forty-eight h after seeding, cells were fixed and stained with a Diff-Quik kit. The number of invading cells was determined by microscopy at a 400x magnification.

### Statistical analysis

Experimental results are shown as mean ± SEM (for variation between sample biological repeats or experiments). The *in vitro* experiments with cultured breast cancer cells, including cell proliferation assay, clone formation assay, invasion assay, morphogenesis assay, and measurements of COX specific activity and cellular ATP levels, were performed with at least biological triplicates. The data were analyzed and compared by paired, 2-tailed Student’s t tests. Multiple comparisons were evaluated using Factorial Analysis of Variance (ANOVA) and proceeded by ad hoc statistical test when necessary. In all cases, statistical tests with p < 0.05 were considered significant.

## Additional Information

**How to cite this article**: Zhang, K. *et al*. COX7AR is a Stress-inducible Mitochondrial COX Subunit that Promotes Breast Cancer Malignancy. *Sci. Rep*. **6**, 31742; doi: 10.1038/srep31742 (2016).
